# The association between family function and adolescents’ non-suicidal self-injury behaviors—is Chinese culture involved?

**DOI:** 10.3389/fpsyt.2025.1522975

**Published:** 2025-08-07

**Authors:** Cheng-jia Yang, Rui Zhou, Yin-Du Liu, Shi-Bin Wang, Cai-Lan Hou, Fu-Jun Jia

**Affiliations:** ^1^ Guangdong Provincial People’s Hospital (Guangdong Academy of Medical Sciences), Southern Medical University, Guangzhou, Guangdong, China; ^2^ The Second School of Clinical Medicine, Southern Medical University, Guangzhou, Guangdong, China; ^3^ Psychiatry/Psychology Department, Guangzhou Red Cross Hospital, Guangzhou, Guangdong, China; ^4^ College of Medicine, Shantou University, Shantou, Guangdong, China

**Keywords:** adolescents, psychiatric disease, non-suicidal self-injury, family function, Chinese culture

## Abstract

**Introduction:**

Non-suicidal self-injury (NSSI) is prevalent among adolescents with psychiatric disorders and has been closely associated with dysfunctional family environments. In the Chinese cultural context, where family structure and attitudes toward sexuality are unique, the link between family function and adolescent NSSI remains underexplored.

**Methods:**

This cross-sectional study recruited 157 adolescent psychiatric patients (aged 13–18) from both outpatient and inpatient settings. Participants were divided into NSSI and non-NSSI groups based on DSM-5 diagnostic criteria. Family cohesion and adaptability were assessed using the Family Adaptability and Cohesion Evaluation Scales II (FACES II), while clinical and sociodemographic data, including sexual orientation, were collected via structured interviews and standardized instruments.

**Results:**

Adolescents in the NSSI group exhibited significantly lower family cohesion (F = 3.92, p = 0.004) and adaptability (F = 2.95, p = 0.001) than those in the non-NSSI group. Binary logistic regression indicated that lower family function, sexual minority status, and higher depression scores (MARDS) were significant predictors of NSSI. “Unknown” sexual orientation also showed a strong association with NSSI.

**Discussion:**

Poor family functioning, particularly in cohesion and adaptability, may increase the risk of NSSI among Chinese adolescents with psychiatric conditions. Additionally, the interplay of Chinese cultural views on family and sexuality may amplify this vulnerability. These findings suggest that family-based interventions and culturally sensitive approaches are critical for the prevention and treatment of NSSI in this population.

## 1 Introduction

Non-suicidal self-injury behavior refers to self-harm behaviors without a suicidal attempt (1). In 2013, the Diagnostic and Statistical Manual of Mental Disorders, Fifth Edition (DSM-5) tentatively listed NSSI as an independent psychiatric condition (2). It has been suggested that NSSI is regarded as one of the behaviors due to negative attributional style, which is one of the influential factors of depression (3–5). Meanwhile, individuals who engaged with NSSI reported far higher depression levels than those of non-NSSI ones (6–8). In addition, several cross-sectional and longitudinal studies suggested that non-suicidal self-injury behavior was one of the strongest predictors of suicidal attempts (9–13). Compared to the non-NSSI patients diagnosed with depression, patients of depression engaged in NSSI had significantly 4.8 times higher odds of suicide attempts (9). Moreover, 17% to 18% of community adolescents had NSSI (14, 15), and for adolescent psychiatric patients the incidence was 50%–70% (16, 17). Obviously, NSSI among adolescents is now a significant social health issue (18).

The majority of adolescents who engage in NSSI seem to have deficiency in family function. Nock established a widely accepted model on the occurrence and maintenance of NSSI in 2006 (1). In this model, a malfunctioned family can cause interpersonal and internal vulnerabilities (such as childhood maltreatments) that make it difficult for adolescents to deal with stressful life events appropriately—those who failed to effectively express the need for help may vent their depression by NSSI.

Thereinto, family exerts impacts on NSSI, both directly and indirectly (19–21) (22–24). Different NSSI studies in Taiwan, China (25), Iran (26), and the United Kingdom (27) have all demonstrated a link between family dysfunction and the development of NSSI. In the study, Iranian adolescents with weak family function had a 13-fold higher risk of developing NSSI than those with strong family function. Moreover, parent–child interaction styles, such as high parental emotional expression (criticism) (28, 29), mother’s harsh parenting style (25), and less perceived parental support (30, 31), may directly increase the occurrence of NSSI in adolescent populations. In addition, single-parent family and low family function were also influential factors of NSSI (30–32), which is consistent with the findings about suicidal ideation.

Despite this wealth of the aforementioned important research, their conclusions were somehow inconsistent. The majority of the research reported a negative relationship between NSSI and family function (22–24, 26, 33), yet the two held that there could be positive correlations between them (23, 34). One study found that mother–child cohesion is negatively related to NSSI when both parents worked outside their home, according to a study of NSSI and family cohesion for Chinese left-behind children (23). Accordingly, left-behind adolescents who have better mother–child cohesiveness would experience more unmet parenting expectations than those who have less cohesion, which will, in turn, raise negative emotions and the risk of NSSI behaviors (23). The other discovered that some “perfectionists” who have a good parent–child relationship with their parents may feel additional pressure to achieve their parents’ standards, which may result in the formation of NSSI behaviors (34). In addition, recent studies found no significant relationship between family-related factors and NSSI after controlling proper variables (35). We suspect that these heterogeneities may be attributed to the Chinese culture or subcultures of Chinese families. The concept of family function varies cross-culturally. Studies in multiple countries have discussed cultural adaptations (36–42) (43, 44). Thus, emphasizing Chinese family culture in research is essential.

Regrettably, few studies have examined what effects Chinese culture may have on these adolescents. Though the concept of “family” appears to be fundamental, the difference between Eastern and Western cultures makes it necessary to be clarified in the Chinese context. Here we believe that Chinese culture will have an impact on understanding NSSI among Chinese adolescents. So, it is meaningful to repeat some of the investigations in mainland China. Sexual minority populations include individuals who identify as lesbian, gay, bisexual, queer, or questioning (45). Even though a considerable amount of literature in this field has historically centered on adults, it is crucial to acknowledge that adolescence remains a pivotal stage in an individual’s life. This stage is characterized by substantial physical, emotional, and social transformations. For sexual minority youth, these changes introduce heightened intricacies and challenges. Often, they are grappling with identity construction and societal prejudice during this critical period, making their journey even more complex and difficult (45).

Two themes in Chinese culture were of interest: the concept of family and the perception of sexuality in China. Family is the fundamental unit of Chinese culture, whereas the perception of sexuality serves as its organizational framework.

While the western family structure is typically a nuclear family consisting of two biological parents and their child(ren) all in one household dwelling, the Chinese family is traditionally depicted as highly cohesive units with three-generational household (46, 47); the Chinese traditional family culture permeates through the life trajectories of Chinese people and deeply influence their mental status. Fei Xiaotong, a sociologist on Chinese culture, claims that, in contradiction to bunches of straws that are clearly distinct from each other, the Chinese family model appears to be ripples, with an individual at the center. The individual’s own social relevance and relatives at different levels of kinship constitute layers of the circles with differing degrees of influence on the individual (48). As a result, the concept of “family” is fluid in Chinese culture context. Away from the center of ripples are relatives in different outskirts exerting social influence somewhere in the life trajectory of the individual to varying extents. This sequential pattern shapes the Chinese concept of “family” (or “家庭/jia-ting”) (48).

The perception of sexuality serves as the organizational framework of Chinese family: in order to maintain emotional stability across the aforementioned ripple circles, Chinese culture emphasizes same-gender lineage bonding (e.g., keeping in touch with cousins or siblings of the same gender), which extends into a man and his wife’s married lives as a way for each of them to maintain cohesion with their extended families (48). Same-gender relationships are somehow viewed as important components of lineage, and this organizational framework, named as “jia-zu (家族)”, encourages many same-gender interactions (48). Excessive homosexual contact, however, is frowned upon in Chinese culture (49). A research conducted in southern China revealed that Chinese parents who have the power to affect their children’s marriage decisions often rejected same-sex partnerships (50)—homosexuality is considered inappropriate in Chinese society (49, 50). In sum, Chinese culture has a dialectical view of same-gender relationships, and it is a concern that this ambivalence may contribute to family dysfunction and adolescents’ mal-development in mental status and behaviors.

Based on the aforesaid family structure, family function refers to the interpersonal interactions, family communication, family activities, shared decision-making, and family adaptability (51), and it bears the influence of Chinese culture. Specifically, interpersonal communication is an important aspect of family function that can promote adolescents’ social skills and emotional development. Good family communication can help adolescents better express their feelings and needs, thereby reducing their anxiety and tension. The open and transparent communication between family members makes them more likely to share their problems with family members and seek help and support. More family activities can help adolescents better understand family members and form healthy family relationships, which can reduce their negative emotions and behavioral problems. Joint decision-making can promote adolescents’ sense of responsibility and self-worth, which helps adolescents better understand the complexity of things and learn to weigh the pros and cons. Family adaptability can help family members better adapt to external environmental changes and enhance their self-control and resilience (51).

Therefore, the aim of this study was to investigate the correlation between family function and NSSI based on adolescent psychiatric patients, which may provide evidence for family support strategies in clinical psychiatry. We have two hypotheses, the first is that poor family function will lead to psychological disorders and thus self-injurious behaviors, and the second is that perceptions of sexuality will have an impact on the correlation between family function and mental health in the Chinese cultural context.

## 2 Methods

### 2.1 Study design and participants

The study was conducted from October 2020 to December 2020. All interviews were conducted by professional investigators including experienced psychiatrists and counselors. Every participant was chosen from one of the big general hospital’s outpatient or inpatient units. All of the participants were interviewed and assessed face to face. Patients with mental disorders aged between 12 and 17 would be included in the study. Those with psychotic symptoms, reluctance to engage, and difficulty cooperating during the interview would be excluded from the research.

We used the DSM-5 diagnostic criteria of NSSI (2): the patients who purposefully injured their skin’s surface without intending to commit suicide five times or more for the previous year were allotted into the NSSI group, while the patients who never engaged in any form of self-injury were allotted into the non-self-injury (NSI) group.

The hospital’s Ethics Committee for Clinical Research approved the research proposal. Informed consent was obtained from all participants and their guardians to participate in the interview and evaluation. The participants can withdraw from the interview at any time without any reason.

### 2.2 Assessments

#### 2.2.1 Sociodemographic assessment

The sociodemographic data include gender, age, ethnicity, place of residence, sexual orientation, educational years, occupation, and body mass index (BMI, kg/m^2^) (52). They were collected in the form of self-filled questionnaires.

Since it is considered improper in Chinese culture to have profound discussions regarding adolescents’ sexual orientation (see “Introduction”), we limited the inquiry to only three categories. Sexual orientation was designed as “heterosexuality”, “sexual minority”, or “not sure”. “Not sure” means “I have no idea about sexual orientation” or “I don’t know what my sexual orientation really is”.

#### 2.2.2 NSSI assessment

The NSSI was assessed with a revised Adolescent Self-Harm Scale and an unstructured case-by-case interview. Regarding the Adolescent Self-Injury Scale, developed by Zheng Ying in 2006 and revised by Feng Yu in 2008 (53), its score has good reliability and validity, indicating good homogeneity. The scale consists of 19 ways of NSSI. We used the scale for a comprehensive screening of NSSI. The scale consisted of two parts, one of which assessed the frequency of one way of NSSI, and the other of which assessed the severity of the NSSI ways. Both parts were scored with a Likert five-point scale, ranging from 0 to 4. Those participants scored 0 on the two scale parts were included in the non-NSSI group, while the ones scored more than 0 were considered likely to meet the diagnosis of NSSI and were included in a further evaluation. The additional evaluation was conducted based on the DSM-5 diagnosis criteria of NSSI, including (1) have you performed NSSI more than five times in the past year, (2) have you ever had a suicidal intent, (3) do you have a history of self-injury, and (4) other problems related to the DSM-5 diagnostic criteria of NSSI (53).

#### 2.2.3 Family relationship and function assessment

As emphasized in the “Introduction”, the Chinese culture serves as a framework for the concepts and metrics of family relationships and function.

Family relationship was assessed as qualitative data by face-to-face interviews with the participants and their parents. Several questions about their parent–child relationship, parents’ marital affection status, and siblings’ relationship were asked in detail. Questions like “do you feel satisfied with the relationships between you and your parents/the relationships between your parents/the relationship with you and your siblings” and “do the disagreements and conflicts often occur in your family and how do your family members resolve the problems” were asked during the interviews. Parent–child relationships, siblings’ relationships, and marital affection of the parents were classified into three categories: bad, fair, and good, respectively (see below and **Table 1**).

**Table 1:** Family function assessment of the youth with and without NSSI.

	NSSI (N=80)		Non-SI (N=77)		Total (N=157)		Statistics		
	n	%	n	%	n	%	χ2a	df	P
Parent-child Relationship							7.18	2	0.02
Bad	19	24	8	10	27	17			
Fair	31	39	26	34	57	36			
Good	30	38	43	56	73	46			
Marital Affection of Parents							3.27	2	0.19
Bad	18	23	9	12	27	17			
Fair	27	34	31	40	58	37			
Good	35	44	37	48	72	46			
Siblings Relationship							4.77	3	0.15
Bad	7	8.75	2	2.59	9	5.73			
Fair	22	27.5	15	19.4	37	23.5			
Good	22	27.5	26	33.7	48	30.5			
Singleton	29	36.2	34	44.1	63	40.1			
	mean	SD	mean	SD	mean	SD	T/ F^b^	df	p
Family Cohesion	1.34	0.61	1.70	0.90	1.49	0.50	3.92	155	**0.004**
Family Adapt Ability	1.80	0.89	2.39	0.98	1.49	0.50	2.95	155	**0.001**

Bold values: *P* < 0.05.

NSSI, non-suicidal self-injury behavior; SI, self-injury.

a Pearson chi-square tests.

b Independent-samples *t*-test or one-way analysis of variance.

“Bad” refers to a relationship that is marked by disagreements, frequent inability to come to an agreement (most days over a 1- to 2-year period), limited time for meaningful discussion, and, in certain situations, even violent confrontation.

“Fair” refers to situations where there are disagreements between family members, and these disagreements can be sometimes settled through a joint effort from the family members, although sometimes they cannot be resolved for a long time.

“Good” refers to a situation in which there are generally a few disputes between family members, and when there are, they can be resolved via a joint effort. Positive interactions also occur often between the family members.

Family function assessment was conducted through the Family Adaptability and Cohesion Evaluation Scales (FACES II) (51). FACES II is a scale for evaluating family functions, compiled by Olson in 1982. The scale shows good internal consistency (Cronbach’s alpha = 0.943) (54).

It is a self-report scale that evaluates two aspects of family function: (1) cohesion, which is the strength of the bonds of emotion among family members and (2) adaptability, the ability of the family system to change in response to situational and developmental stress. There are 30 items, covering family communication, relationships, joint activities, joint decision-making, housework, and family adaptability. The test–retest reliability of the two parts was 0.84 and 0.54, and the internal consistency was 0.85 and 0.76, respectively (54). The scores range from 28to 92 for cohesion and 22–70 for adaptability. The higher the score, the greater the family cohesion or adaptability is (51).

Then, we converted the cohesion scores and the adaptation scores into two sets of hierarchical variables based on their raw values (54). Each of the four intervals of the raw cohesion scores—below 55, 55–63, 64–72, and beyond 72—was given a new score from 1 to 4, referring to four intervals: “entanglement”, “intimacy”, “freedom”, and “looseness”. Similarly, the raw adaptation score was divided into four intervals, too—beyond 57, 51–57, 44–50, and below 44, and they were newly given a score from 1 to 4, referring to “irregular”, “flexible”, “regular”, and “rigid” (54).

#### 2.2.4 Clinical evaluation

Clinical evaluation included the MINI-International Neuropsychiatric Interview (M.I.N.I.) (55), psychopathological evaluation, medical history collection, and psychiatric drug information. Details on the type of psychiatric drugs were collected, including antidepressants, antipsychotics, anticonvulsants, and benzodiazepines. Doses of antipsychotic drugs were converted into the prescribed daily dose/defined daily dose ratio (PDD/DDD ratio) (56).

The Mood Disorder Questionnaire (MDQ) (57), the Montgomery–Asberg Rating Depression Scale (MARDS) (58), the Young Mania Rating Scale (YMRS) (59), and the Brief Psychiatric Rating Scale (BPRS) (60) were chosen to assess the mental state of the participants.

#### 2.2.5 The Mood Disorder Questionnaire

The MDQ was used to assess subthreshold mood bipolarity. It is composed of 13 questions that inquire about potential (hypo)manic symptoms. Its Cronbach’s alpha, test–retest reliability, and the content validity were 0.83, 0.76, and 0.80, respectively (61). A score of 7 or above is used for the identification of bipolar disorder.

#### 2.2.6 The Montgomery–Asberg Rating Depression Scale

Each item on the scale can have a score between 0 and 6, for a total score range of 0 to 60 for the 10 things. The “depressive state” that this scale measures relates to the epidemiologic definition of depression rather than a clinical diagnosis (58). Its Cronbach’s α reported in a recent study was 0.70 (62). A higher MARDS score suggests a more severe depression.

#### 2.2.7 The Young Mania Rating Scale

The 11-item Young Mania Rating Scale (YMRS) was used to assess the symptoms of mania (59). One prior study has demonstrated good reliability and validity of the scale (63). A higher score on the YMRS indicates more severe mania symptoms.

#### 2.2.8 The Brief Psychiatric Rating Scale

The BPRS assessed psychopathology severity. A cumulative mean score between 1 and 7 is produced by rating each of the 18 items on a scale from 1 (not present) to 7 (severe). For the BPRS total score, one previous work has proved that its reliability is moderate [intraclass correlation coefficient (ICC), 0.54] (64).

All interviews were conducted individually, with each lasting 30–50 min. The trained staff collected information by questioning the respondents face-to-face, based on the MINI, the MARDS, the YMRS, and the BPRS. Socio-demographic information, FACES II, and the MDQ were self-reported.

### 2.3 Statistical analyses

All data is analyzed by software SPSS22.0. Independent sample *t*-test and chi-square tests were applied to compare the differences between NSSI and non-NSSI groups in sociodemographic, family function, and clinical characteristics. Binary logistic regression analysis was used to explore the risk factors of developing into NSSI. For all statistical analyses, two-tailed *P*-value <0.05 was considered statistically significant in this study.

## 3 Results

A total of 164 adolescent psychiatric patients were recruited, in which seven did not complete the self-assessment of the scales. Finally, 157 completed the review and evaluation, which were included in the final analysis. The response rate was 95.7%. All of the participants were divided into NSSI group (*n* = 80) and non-NSSI group (*n* = 77).

**Table 2** shows the sociodemographic and clinical characteristics of the participants. The mean age of the NSSI group was 15.4, while it was 15.34 for the non-NSSI group. Moreover, 73.8% of the participants were diagnosed with mood disorder, and 11.2% of them were diagnosed with neurotic, stress-related, or somatoform disorders. There were more patients diagnosed with mood disorders in the NSSI group than in the non-NSSI group (*χ* ^2^ = 6.53, *P* = 0.03). The NSSI group was characterized as being with more comorbidity (*F* = 2.45, *P* = 0.015), with a lower percentage of patients whose current disease episode was their first (*χ* ^2^ = 4.37, *P* = 0.036), with a higher MARDS score (*F* = 3.67, *P* < 0.001) and BPRS score (*F* = 2.12, *P* = 0.01), and with a higher dose of antipsychotics (*F* = 3.58, *P* < 0.001), benzodiazepines (*F* = 3.46, *P* = 0.001), and anticonvulsants (*F* = 2.45, *P* = 0.015). Compared with the non-NSSI group, there were more sexual minority and fewer unknown orientations in the NSSI group (*χ* ^2^ = 11, *P* = 0.004).

**Table 2:** Sociodemographic and clinical characteristics of the youth with and without NSSI.

	NSSI (N=80)		Non-SI (N=77)		Total (n=157)		Statistics		
	n	%	n	%	n	%	χ^2 a^	df	P
Male	17	21	26	34	42	27	3.00	1	0.07
Han Chinese	78	98	74	96	152	97	0.24	1	0.61
Urban Area	70	88	64	83	134	85	0.60	1	0.43
Sexual Orientation							11.00	2	**0.004**
Heterosexuality	53	66	47	61	100	64			
Sexual Minority	23	29	13	17	36	23			
Unknown	4	5	17	22	21	13			
Psychiatric Diagnosis							6.53	2	**0.03**
Mood disorder	66	83	50	74	116	74			
Neurotic, stress related and somatoform disorders	9	11	15	20	24	15			
Others	5		12		17	11			
First-episode	42	53	53	69	95	61	4.37	1	**0.036**
Psychiatric Family History	10	13	10	13	20	13	0	1	0.92
	mean	SD	mean	SD	mean	SD	T/F ^b^	df	P
Age (years)	15.4	1.75	15.34	1.88	15.39	1.81	0.38	155	0.69
Educational Year	9.63	1.81	9.60	1.73	9.61	1.76	0.09	155	0.092
BMI	20.9	4.38	20.66	3.38	20.80	3.91	0.42	155	0.67
Hospitalizations	0.80	0.89	0.36	0.60	0.59	0.79	3.57	155	**<0.001**
Comorbidity Number	1.66	1.13	1.22	1.09	1.44	1.13	2.45	154	**0.015**
MDQ	1.53	0.50	1.62	0.48	1.57	0.49	1.24	155	0.21
MARDS	20.5	9.62	14.82	9.91	17.74	10.1	3.67	155	**<0.001**
YMRS	3.14	5.25	2.10	2.76	2.63	4.24	1.53	155	0.12
BPRS	29.30	7.75	26.7	7.37	28.0	7.65	2.12	155	**0.01**
Antipsychotics	0.49	0.73	0.16	0.36	0.33	0.60	3.58	155	**<0.001**
Antidepressants	1.02	1.14	0.79	1.03	0.91	1.09	1.28	155	0.201
Benzodiazepines	0.19	0.28	0.05	0.17	0.12	0.24	3.46	155	**0.001**
Anticonvulsants	0.55	0.72	0.23	0.48	0.39	0.63	3.19	155	**0.002**

Bold values: *P* < 0.05.

BMI, body mass index; BPRS, Brief Psychiatric Rating Scale; MARDS, Montgomery–Asberg Depression Rating Scale; MDQ, Mood Disorder Questionnaire; NSSI, non-suicidal self-injury behavior; SI, self-injury behavior; YMRS, Young Mania Rating Scale.

a Pearson chi-square tests.

b Independent-samples *t*-test or one-way analysis of variance.

**Table 1** shows the assessment of family in both NSSI and non-NSSI groups, composed of assessment of relationship between different family members and assessment of family function including family cohesion and family adaptability. Only the parent–child relationship was significantly different between the NSSI and non-NSSI group (*χ* ^2^ = 7.18, *P* = 0.02). There were more “fair” and “bad” parent–child relationships dictated in the NSSI group. Both groups possessed good sibling relationship and good marital affection of parents. As for family function, both groups were equipped with weak family function. The difference was that in terms of whether family cohesion (*F* = 3.92, *P* = 0.004) or family adaptability (*F* = 2.95, *P* = 0.001), the NSSI group had a lower score for both than the non-NSSI group obviously.

**Table 3** shows the result of regression analysis among family function with all of the significant risk factors mentioned above. In the model including family cohesion, the regression analysis suggested a significant association between non-NSSI and a higher level of family cohesion (OR: 0.56, 95% CI: 0.366–0.859), a lower score of MARDS (OR: 1.06, 95% CI: 1.011–1.013), and less sexual minority (OR: 3.76, 95% CI: 1.035–13.71) and unknown sex orientation (OR: 7.31, 95% CI: 1.735–30.84). The model included family adaptability, and it showed a similar result. The non-NSSI group had a higher level of family adaptability (OR: 0.57, 95% CI: 0.341–0.959), a lower score of MARDS (OR: 1.07, 95% CI: 0.011–1.134), and less sexual minority (OR: 3.96, 95% CI: 1.115–14.07) and unknown sex orientation (OR:6.86, 95% CI:1.6 71-28.18).

**Table 3:** Sociodemographic, clinical, and family risk factors of developing into NSSI groups (two binary logistic regression analysis).

	NSSI vs. Non-SI		
	P	Odds ratio*	95%C.I.
Including Family Cohesion			
Sex Orientation (self-reported)			
Heterosexuality	**0.025**		
Sexual Minority	**0.044**	3.76	1.035-13.71
Unknown	**0.007**	7.31	1.735-30.84
MRADS	**0.026**	1.06	1.011-1.013
Family Cohesion	**0.008**	0.56	0.366-0.859
Including Family Adapt Ability			
Sex Orientation			
Heterosexuality	**0.028**		
Sexual Minority	**0.033**	3.96	1.115-14.07
Unknown	**0.008**	6.86	1.671-28.18
MRADS	**0.020**	1.07	1.011-1.134
Family Adapt Ability	**0.034**	0.57	0.341-0.959

Bold values: *P* < 0.05. The impact of psychiatric diagnosis, comorbidity number, hospitalization times, Brief Psychiatric Rating Scale, antipsychotics, benzodiazepines, and anticonvulsants have been controlled in the analysis and have not been represented in the table.

MARDS, Montgomery–Asberg Depression Rating Scale; NSSI, non-suicidal self-injury behavior; non-NSSI, do not have non-suicidal self-injury behavior.

* An odds ratio equal to 1.0 means that both compared values of the given parameter yield identical odds of influence; likewise, an odds ratio of higher than 1 indicates greater odds of influence, vice versa.

## 4 Discussion

The investigation came to a few key conclusions. Adolescent psychiatric patients with NSSI had a lower level of family cohesion and adaptability than those without NSSI. In addition, the sexual orientation and depressive symptoms of adolescents in the NSSI group were also found to be significantly different from those in the non-NSSI group.

In this hospital-based study, a low level of family function indicated the occurrence of NSSI (see **Table 3**), which was consistent with most of the previous community-based studies. The study among high school students (*n* = 1,989) in China (25) showed that poor family function predicted the occurrence of NSSI and that this effect may be related to the adolescents’ avoidance/emotion-centered coping strategies. Similar conclusions were drawn from the study of Iranian high school students (*n* = 4216) (26), indicating that adolescents with weak family psychological function were 13 times more likely to experience NSSI than those who had strong function. Furthermore, Cassels M found that the factor of poor family function at age 14 mediated the association between childhood family adversity before age 5 and the occurrence of NSSI between ages 14 and 17, according to a 3-year follow-up of 14-year-old British adolescents (27). In addition, their concealment of NSSI may weaken the connection between NSSI and family function in studies. According to a research by Victor Buitron (65), adolescent NSSI was only significantly associated with poor parental warmth and perceived burden when it appeared to be present at high levels; this association was not significant at low levels of NSSI. In this study, all participants were psychiatric patients and had more severe NSSI.

In the perspective of sexual minority and higher NSSI risk, previous research findings are generally consistent with the results of this study. As shown in **Table 2**, the proportion of “sexual minority” among NSSI practitioners is higher than that among those without self-injury, while the “unknown” proportion is lower. The logistic regression (**Table 3**) indicated that the family function and sex orientation were both individual associating factors of NSSI. Another study shows that bisexual people had up to six times the odds of engaging in NSSI compared to other sexualities (66). Another study explicitly proposed that people who identify as sexual minorities are at an increased risk for suicide. Moreover, the absence of a notable interaction effect suggests that the severity of NSSI does not enhance the influence of sexual orientation on suicide risk; it instead forecasts an equivalent level of heightened risk across all orientations (67). This can be explained by the Minority Stress Theory (66) and “the concealment phenomenon” (68) (69). According to the Minority Stress Theory, sexual minorities face more interpersonal pressures and internalized homophobia, which may lead to a higher NSSI rate (66). The dominating “unknown” percentage within the non-SI group could reflect a degree of concealment: hiding their sexual orientation may serve as a coping strategy toward internalized homophobia (68).

It is suggested that the concept of homo-sex relationships in indigenous Chinese context may be distinct from the homosexual concept in Western cultures, and both concepts exert influence on minority stress in modern China. Actually, the stress surrounding homosexuality emerged in late-20^th^-century China following the introduction of Western psychiatric views which once pathologized homosexuality, and despite its removal from the diagnostic criteria in 2001, the notion endures (70). Comparatively, in pre-modern China, homo-sex relationships were acceptable if they met family obligations of marriage and procreation (see the “Introduction”) (70).

Lastly, in line with the findings of earlier investigations, the NSSI group displayed more severe depressed symptoms. Nock (71) believed that some adolescents may utilized NSSI as a maladaptive coping style to deal with their negative emotion (72). In addition, NSSI was associated with negative attributional styles, which was another important influence of depression. A longitudinal NSSI follow-up study indicated a substantial link between depressed symptoms and NSSI (3), among which negative attributional style was largely consistent with the longitudinal trajectory of NSSI, which was largely congruent with the findings of Hankin and Abela (5). Negative attributional style may also be associated with despair about the future and low confidence in one’s ability to improve the current situation, which was one of the high-risk factors for NSSI (4).

Overall, considering the uniqueness of Chinese culture, we recommend clarifying the distinction between same-sex familial relationships within Chinese family structures and same-sex relationships among sexual minorities before providing psychological counseling to adolescent sexual minorities. It is also essential to confirm the potential sexual minority status rather than homo-sex relationships within a Chinese familial context. Additionally, in contrast to Western families, psychological therapy for Chinese families may necessitate an analysis of extended family members beyond the nuclear family.

## 5 Limitations and strengths

Although these results were informative, some limitations of this study should be noted. First, the present study was a cross-sectional study and failed to show a causal relationship between family function and NSSI. Second, this study only investigated family function data from adolescents and did not further collect data from parents’ perceptions of family function to exclude the impact of adolescents’ valuation on family function in the context of mental disorders. Third, though the FACES has been updated to the fourth edition, its new versions with good reliability and validity have not been translated into Chinese, which limited their use in our study. In addition, this study failed to quantify the severity of NSSI to further explore the correlation between family function and NSSI. However, despite these limitations, the results of this study advanced the scientific and clinical understanding in this area especially in the clinical adolescent population and in the Chinese context. Finally, relying solely on teenagers’ self-assessments of family dynamics may introduce potential biases. Due to the anonymization of sexual minorities, it may lead to concealment behaviors among adolescent NSSI patients (73). Future research is needed to further explore causal relationships between family functioning and NSSI.

## 6 Conclusion

In conclusion, adolescents with worse family function were more likely to develop NSSI, and improving family function may be one of the complementary treatment strategies for NSSI in adolescent psychiatric patients. The characteristics of sexual orientation and the effective response to depressive symptoms also needed to be considered.

## Data Availability

The raw data supporting the conclusions of this article will be made available by the authors, without undue reservation.
